# The impact of secondary hyperparathyroidism on the efficacy of antiresorptive therapy

**DOI:** 10.1186/1471-2474-13-244

**Published:** 2012-12-10

**Authors:** Gyöngyvér Kincse, József Varga, Péter Somogyi, Péter Szodoray, Péter Surányi, János Gaál

**Affiliations:** 1 Department of Rheumatology, ‘Kenézy Gyula’ Hospital, Bartók Béla út 2-26, H- 4032, Debrecen, Hungary; 2 Department of Nuclear Medicine, University of Debrecen, Medical & Health Sciences Centre, Debrecen, Hungary; 3 Department of Orthopedic Surgery, University of Budapest, Budapest, Hungary; 4 Institute of Immunology, Rikshospitalet, Oslo University Hospital, Oslo, Norway

**Keywords:** D vitamin status, PTH level, Antiresorptive therapy, Efficacy

## Abstract

**Background:**

The aim of the present study was to assess whether the efficacy of bisphosphonate treatment is influenced by PTH levels measured in newly diagnosed osteoporotic patients and to identify the threshold value, beyond which PTH level negatively influences therapeutic efficacy.

**Methods:**

One hundred and thirty-eight osteoporotic patients were enrolled into the study. All subjects underwent laboratory screening, bone densitometry with DEXA, and x-ray imaging. The changes in bone density were evaluated after a mean follow-up period of 13.37 ± 1.29 months. Correlation analysis was performed on the clinical data of patients, the percentage changes of BMD values, and the PTH levels measured at the beginning of study, using SPSS software.

**Results:**

The mean age of the subjects was 64.82 ± 10.51 years, and the female-to-male ratio was 116/22. Baseline BMD value measured with AP DEXA scanning was 0.854 ± 0.108 g/cm^2^ in the L_1-4_ vertebrae and 0.768 ± 0.115 g/cm^2^ in the left femoral neck. By the end of the follow-up period, these values changed to 0.890 ± 0.111 g/cm^2^ and 0.773 ± 0.111 g/cm^2^, respectively. We found a statistically significant, negative correlation between PTH levels and the percentage changes of lumbar BMD values measured at the end of the follow-up (correlation coefficient R^2^ = 0.121, p < 0.0001). The analysis of frequency histograms suggested that negative effects on bone might be expected above a PTH level of 60 pg/mL (7.3 pmol/L).

**Conclusion:**

Our findings imply that a baseline PTH level over 60 ng/mL can reduce the efficacy of bisphosphonate treatment.

## Background

Osteoporosis is considered a public health problem in an increasing number of countries. According to the initial results of the epidemiological studies conducted in Hungary during the mid-90’s, primarily in international co-operation, the nationwide prevalence of osteoporosis in the population over 50 years of age was 32.3% among women and 23.6% among men [1]. This means that osteoporosis afflicts approximately 600,000 women and more than 300,000 men overall in Hungary [2].

Anti-resorptive agents, including oral or parenteral aminobisphosphonates (e.g. alendronate, risedronate, ibandronate, and zolendronate), with a mechanism of action based on the blockade of mevalonate metabolism, play an outstanding role in the management of osteoporosis. These agents inhibit the isoprenylation of small GTPases, which are signalling proteins important in the maintenance of cell structure [3].

Calcium and vitamin D sufficiency is a prerequisite to effective antiresorptive therapy, especially in the elderly population [4]. Low vitamin D_3_ level triggers the compensatory release of parathormone (PTH) [5], which increases the risk of fractures [6], as well as interference with neuromuscular function [7,8]. Vitamin D sufficiency, as well as optimal levels of activated vitamin D_3_ (1,25-dihydroxy-vitamin D) can suppress PTH secretion [9]. Above-normal PTH levels were detected in 17.4% of postmenopausal women treated for osteoporosis [10], and a negative correlation was found between PTH and vitamin D levels [11]. When 25(OH)-vitamin D level decreases below 4.6 ng/mL, serum PTH level reaches the upper limit of the normal range (65 pg/mL) [12].

There were some important premises that have prompted us to conduct this study. According to the Bhattoa et al., serum 25(OH)-vitamin D_3_ is below normal in 56.7% of postmenopausal women in Hungary [13], and a significant portion of these patients expectedly have higher than normal PTH levels. Unfortunately, bone loss continues despite adequate bisphosphonate therapy and supplementation with calcium and vitamin D_3_ in 8 to 23% of osteoporotic patients (bisphosphonate resistance) [14], and several publications have suggested a role for secondary hyperparathyroidism in the aetiology of bisphosphonate resistance [15,16].

The aim of the present study was to assess the prevalence of secondary hyperparathyroidism (vitamin D_3_ deficiency) among newly diagnosed osteoporotic patients and to evaluate whether baseline levels of PTH influence the efficacy of anti-osteoporotic treatment (with bisphosphonates) in this population. Furthermore, we determined the threshold, beyond which PTH level has a negative impact on the efficacy of bisphosphonate treatment.

## Methods

### Type of study

This study was a prospective, observational, non-interventional study.

### Inclusion and exclusion criteria

Patients meeting the following criteria were eligible for inclusion: *i)* diagnosed idiopathic osteoporosis with a lumbar and/or femoral T-score lower than −2.5; *ii)* patients newly identified and enrolled to follow-up; *iii)* patients with laboratory findings available.

The exclusion criteria were as follows: *i)* diagnosed secondary osteoporosis; *ii)* history of a malignancy; *iii)* renal failure (GFR <65 mL/min according to the Cockroft-Gault formula); *iv)* severe liver disease; *v)* hypo-/hyperthyroidism; *vi)* malabsorption syndrome; *vii)* hypercalcaemia; *viii)* hypocalcaemia; *ix)* history of renal calculosis; *x)* previous anti-osteoporotic therapy with bisphosphonates, selective estrogen-receptor modulators (SERMs), strontium ranelate, teriparatide, or calcitonin.

### Study population

Two hundred and thirty-two patients met the inclusion criteria, and data from 138 (116 women and 22 men, with a mean age of 64.82 ± 10.51 years and between 43 and 81 years) were available at the time of the ebd of the study. Written informed consent for participation was obtained from each patient. Ninety-seven patients received alendronate, 19 risedronate, 7 zolendronate, and 15 ibandronate. At baseline, 13 patients had a prevalent vertebral, and 59 a prevalent non-vertebral fracture, while 22 had multiple fractures (Table 1.)

**Table 1:** The baseline characteristics of the sudy population

Number of patients	138
Women/men	116/22
Mean age (years)	64.82 ± 10.51
Treatment	Alendronate:97
	Risedronate: 19
	Zolendronate: 7
	Ibandronate:15

The study protocol was approved by the Regional Research Ethics Committee of the Univeristy of Debrecen, Medical and Health Science Center.

### Study endpoints

The primary endpoint of the study was the change of bone mineral density (BMD) values (and T-scores) during one year with appropriate combination therapy with a bisphosphonate, vitamin D_3_, and calcium.

### Implementation of the study

At baseline, the patient’s medical history was taken, and physical examination was performed. All subjects underwent bone densitometry of the lumbar spine (L_1-4_ vertebrae) and of the left femoral neck with antero-posterior dual-energy x-ray absorptiometry (AP DEXA) scanning, as well as an x-ray of the dorsal-lumbar spine. Laboratory screening comprised the following: erythrocyte sedimentation rate (ESR), C-reactive protein (CRP), complete blood count (CBC), serum calcium and phosphate, blood urea nitrogen (BUN), creatinine, aspartate aminotransferase (AST), alanine aminotransferase (ALT), alkaline phosphatase, serum albumin, urinary calcium/creatinine ratio, thyroid stimulating hormone (TSH), intact parathyroid hormone (iPTH), osteocalcin, carboxy-terminal collagen crosslinks (CTX).

Serum PTH was measured using electrochemiluminescence immunoassay (Roche Diagnostics GmbH, Mannheim, Germany), where the inter-assay CV was <7%. This assay employs a sandwich test principle in which a biotinylated monoclonal antibody reacts with the N-terminal fragment (1–37), and a monoclonal antibody labeled with a ruthinium complex reacts with the C-terminal fragment (38–84). The antibodies in this assay are reactive with epitopes in the amino acid regions 26–32 and 37–42.

We repeated the DEXA scan after a one-year (13.37 ± 1.29 months) follow-up on the average, and recorded the number of incident bone fractures and cases of renal stone formation that had occurred du-ring this period.

The subjects received adequate bisphosphonate therapy with alendronate, risedronate, or ibandronate administered in combination with 6000 IU/week vitamin D_3_, as well as 1000 mg/day calcium during the follow-up period.

The patients’ compliance was evaluated at the scheduled quarterly visit, and a more than 80% compliance was acceptable to continue the study.

### Statistical analysis

We formed two subgroups based on the change in bone mineral density (BMD) values as a result of therapy. The group of responders included all those patients in whom BMD level decreased as compared to the baseline value; the rest of the patients formed the group of non-responders. The definition of bone loss (non-responders) was made if the bone mineral density was decreased by more than 1% of the initial value, because the variation coefficient is less than 1% in the case of Lunar Prodigy DXA device, which was used in this study. The normality of the distribution of datasets was checked with Kolmogorov-Smirnov test. We compared data from two independent groups using Mann–Whitney test. A general linear model was used to identify the factors influencing the magnitude of the change in bone density of the spine and of the femoral neck. All statistical analyses were performed with version 19.0 of the IBM SPSS Statistics software package.

## Results

On enrolment in the study, PTH levels were normal in 112 patients, while it elevated in 26 cases. Baseline BMD measured at the L_1-4_ vertebrae, and at the left femoral neck by AP DEXA scanning was 0.854 ± 0.108 g/cm^2^, and 0.768 ± 0.115 g/cm^2^, respectively.

After a follow-up period and appropriate therapy for over one year on average, bone density increased to 0.890 ± 0.111 g/cm^2^ at the lumbar spine, and to 0.773 ± 0.111 g/cm^2^ at the femoral neck. Thus, the mean increase in BMD was 0.036 g/cm^2^ and 0.005 g/cm^2^, respectively.

**Figure 1:**
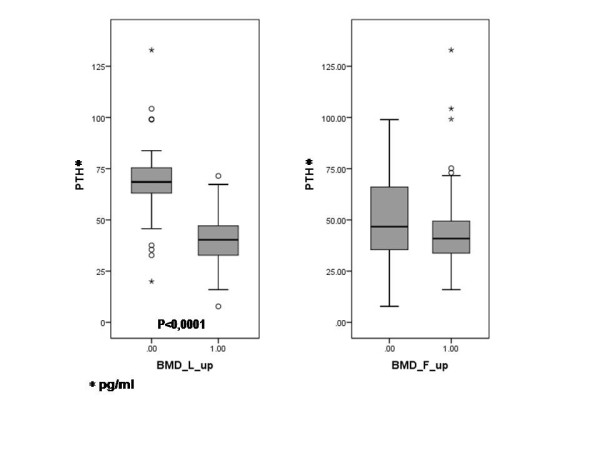
Baseline parathormone levels in the responder and non-responder subsets (0: declining bone mineral density/non-responders/, 1: improving bone mineral density/responders/).

We found a statistically significant (p < 0.0001) difference between the baseline PTH levels of patient subsets with declining or non-declining bone density (using either BMD or T-score as the basis for grouping). However, creating these subsets according to femoral bone density (based on either the BMD or the T-score), the difference between PTH values was no longer significant (Figure 1).

**Figure 2:**
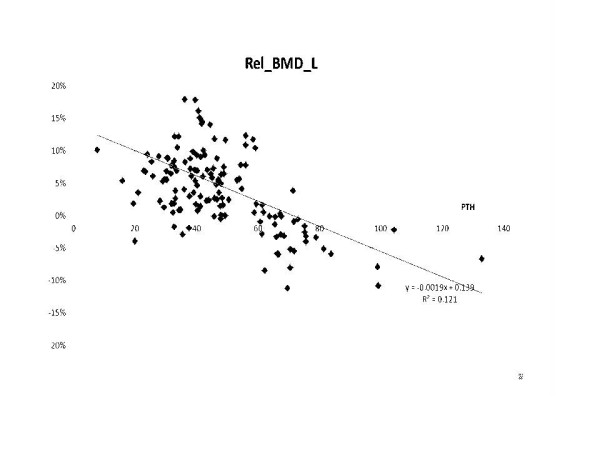
The relationship between baseline parathormone levels, and the changes in lumbar bone mineral density.

The general linear model showed a strong, significant (p < 0.0001) relationship between baseline PTH levels and the relative change in lumbar BMD (Figure 2). A similar correlation could not be demonstrated for the femoral neck, other variables (such as age, gender, etc.), and the change in BMD (p > 0.05).

**Figure 3:**
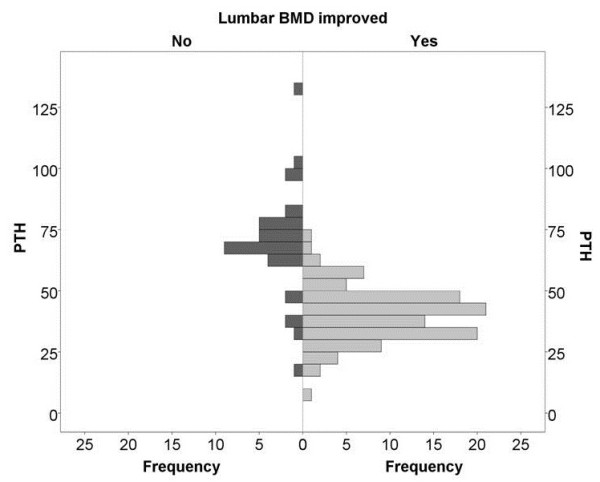
The frequency histogram of the parathormone levels in responders and non-responders.

We determined the prognostically optimal threshold of baseline PTH level, which best differentiated the subsets with declining and improving lumbar BMD from each other. According to the frequency histogram (of PTH level plotted in units of 5 pg/mL), this was at approximately 60 pg/mL for the lumbar spine (Figure 3). Regarding the femoral neck, the frequency histogram did not show any substantial difference between the subsets with improving or declining bone density.

New (incident) vertebral or non-vertebral fractures occurred in one and five subjects, respectively.

## Discussion

Vitamin D_3_ deficiency and accompanying secondary hyperparathyroidism are extremely prevalent in the senior population of developed countries – regardless of geographical location or terrestrial latitude [17]. The weight of this problem is illustrated – among others – by a survey conducted in Austria, the United Kingdom (UK), and Mexico. This study revealed that the proportion of patients receiving calcium and vitamin D_3_ supplementation was 73% in Austria, 15% in the UK, and 12% in Mexico. Furthermore, only 20% of the patients in Austria were taking vitamin D_3_ – where the latter was provided free to osteoporotic patients [18,19].

In 98% of patients hospitalized for non-traumatic fractures, 25(OH) vitamin D_3_ levels were found to be low, which was commonly associated with elevated PTH level and hence, accelerated bone loss [20,21]. An inverse relationship has been shown between the risk of osteoporotic hip fractures and increased vitamin D_3_ intake, as well as greater adherence to supplementation [22].

There is an increasing number of reports suggesting that inadequate (<30 ng/mL) serum 25(OH) vitamin D_3_, and consecutively elevated PTH levels are among the essential factors behind bisphosphonate resistance, leading to unsatisfactory response to bisphosphonates [15,16].

Previous studies have demonstrated that the increase of BMD in every skeletal region is greater in vitamin D-replete patients than in those with vitamin D deficiency. Additionally, the risk of incident osteoporotic fractures is 1.77 times higher in the latter population, than in vitamin D-replete individuals [23].

Our findings confirm that elevated (>60 pg/mL) serum PTH levels exert an unfavourable influence on the efficacy of bisphosphonate therapy. We believe it would be important to determine PTH levels before bisphosphonate treatment is initiated. In particular, similar to a variety of other disorders, such as diabetes and hypertension, the success of anti-osteoporotic therapy is essentially dependent on a specific target PTH level (<60 pg/mL in uor case). From an economic point of view, the cost of a single PTH measurement is far less than the annual expenditure on ineffective bisphosphonate therapy.

It is controversial whether administering an initial, oral loading dose of (40000 to 300000 IU) vitamin D is justified before initiating anti-resorptive therapy in hypovitaminosis D_3_. The administration of a single, 300000-IU dose of vitamin D_3_ could successfully normalize the serum 25(OH) vitamin D_3_ levels of patients within three months, without major adverse effects [24]. Regarding the 30 ng/mL (75 nmol/L) serum 25(OH) vitamin D_3_ concentration as the target level, others have provided therapeutic recommendations on the single loading dose [25].

The fracture-reducing effect of daily supplementation with 800 to 1000 IU oral vitamin D_3_ is evident. In direct contrast to this, others have observed an increase (compared to placebo) in the number of falls, and fractures among their elderly female patients treated with yearly oral mega-dose (500000 IU) of vitamin D_3_[26].

Although the authors could not explain this finding, the need for reducing the excessively high baseline level of PTH has not been fully addressed in previous studies.

Serum PTH levels, the vitamin D_3_-replete state, and supplementation with vitamin D_3_ are of significant importance in osteology. It is highly probable that the higher (>60 pg/mL in our study) baseline PTH levels observed before the initiation of anti-osteoporotic treatment have a clear effect on the therapy outcome. Whether long-term oral vitamin D supplementation or the administration of a single high dose vitamin D_3_ treatment is justified can be determined by additional large-scale studies.

## Conclusion

This is the first study indicating the connection between the treatment efficacy and the PTH levels in newly diagnosed osteoporotic patients. Moreover, we determined a potentially “harmful” cut-off value for PTH levels. Our results suggest that the PTH levels higher than 60 pg/mL have negative prognostic value for the efficacy of antiporotic treatment.

### Study limitations and strength

The relatively low patient number and the short follow-up are the possible limitations of this study. The main strenght of the study is that this is the first study to determine a cut-off PTH value, which affect the success of the bisphosphonate treatment in patients with osteoporosis.

## Competing interest

The authors declare that they have no competing interests.

## Authors’ contributions

GK participated in patient recruitment, data capture and in the design of the study. JV performed the statistical analysis. PS participated also in the recruitment of patients and in the organisation of the study. PS participated in the design of the study and helped in drafting the manuscript. PS participated in the patient recruitment and data acquisition. JG conceived of the study, and participated in its design and coordination. All authors read and approved the final manuscript.

## Pre-publication history

The pre-publication history for this paper can be accessed here:

http://www.biomedcentral.com/1471-2474/13/244/prepub
